# Graph-Gated Relational Reasoning for Enhanced Coordination and Safety in Distributed Multi-Robot Systems: A Decentralized Reinforcement Learning Approach

**DOI:** 10.3390/s25237335

**Published:** 2025-12-02

**Authors:** Tianshun Chang, Yiping Ma, Zhiqian Li, Shuai Huang, Zeqi Ma, Yang Xiong, Shijie Huang, Jingbo Qin

**Affiliations:** 1 Merchant Shipping Academy, Shanghai Maritime University, Shanghai 201306, China; 2 College of Energy and Mechanical Engineering, Shanghai Electric Power University, Shanghai 201306, China; 3 Engineering College, Shanghai Ocean University, Shanghai 201306, China

**Keywords:** Unmanned Surface Vehicle (USV), multi-agent reinforcement learning, cooperative control, transformer, contextual reasoning, multi-modal perception, collision avoidance, maritime safety, maritime robotics

## Abstract

The autonomous coordination of multi-robot systems in complex, environments remains a fundamental challenge. Current Multi-Agent Reinforcement Learning (MARL) methods often struggle to reason effectively about the dynamic, causal relationships between agents and their surroundings. To address this, we introduce the Graph-Gated Transformer (GGT), a novel neural architecture designed to inject explicit relational priors directly into the self-attention mechanism for multi-robot coordination. The core mechanism of the GGT involves dynamically constructing a Tactical Relational Graph that encodes high-priority relationships like collision risk and cooperative intent. This graph is then used to generate an explicit attention mask, compelling the Transformer to focus its reasoning exclusively on entities rather than engaging in brute-force pattern matching across all perceived objects. Integrated into a Centralized Training with Decentralized Execution (CTDE) framework with QMIX, our approach demonstrates substantial improvements in high-fidelity simulations. In complex scenarios with dynamic obstacles and sensor noise, our GGT-based system achieves 95.3% coverage area efficiency with only 0.4 collisions per episode, a stark contrast to the 60.3% coverage and 20.7 collisions of standard QMIX. Ablation studies confirm that this structured, gated attention mechanism—not merely the presence of attention—is the key to unlocking robust collective autonomy. This work establishes that explicitly constraining the Transformer’s attention space with dynamic, domain-aware relational graphs is a powerful and effective architectural solution for engineering safe and intelligent multi-robot systems.

## 1. Introduction

The autonomous coordination of Unmanned Surface Vehicles (USVs) in real-world maritime environments remains a significant technical challenge [1,2]. Effective multi-agent systems must operate under a triad of harsh conditions: (1) severe multi-modal sensor degradation due to factors like water turbidity, sun glare, and acoustic reverberation; (2) unpredictable environmental dynamics, including currents and wave-induced motions that render pre-planned paths infeasible; and (3) safety-critical constraints under fragile and low-bandwidth communication, which makes decentralized execution a necessity.

Existing approaches to multi-USV coordination often fail to adequately address this triad of challenges. Traditional planning methods, such as potential fields or Boustrophedon decomposition, are brittle as they typically assume reliable sensing and static environments [3,4]. While recent Multi-Agent Reinforcement Learning (MARL) methods show promise, standard architectures often suffer from a “perceptual poverty [5].” They typically employ simple function approximators, like Multi-Layer Perceptrons (MLPs), which process flattened observation vectors. This approach lacks the capacity to interpret noisy, conflicting multi-modal sensor streams or to explicitly reason about the dynamic, causal relationships between the agent, its teammates, and surrounding obstacles. This mechanistic limitation is the critical bottleneck, often leading to reactive, suboptimal, and unsafe behaviors when faced with real-world complexity. To address these limitations, we propose a novel framework centered on a concrete architectural innovation for perception and control. Our approach begins at the perception level, employing a dedicated multi-modal fusion pipeline that processes raw, noisy visual and sonar data into a structured, entity-centric representation. This provides a high-quality, robust input for the decision-making core. Recognizing the importance of structured reasoning, other recent studies have also begun to explore hybrid architectures that integrate Graph Neural Networks with Transformers to better capture the complex spatio-temporal relationships inherent in multi-agent systems. Our work builds upon this direction by introducing an explicit gating mechanism, which fundamentally differs from existing approaches.

We introduce the Graph-Gated Transformer (GGT), a novel neural architecture designed to inject explicit relational priors directly into the self-attention mechanism for multi-robot coordination. The core mechanism of the GGT involves dynamically constructing a Tactical Relational Graph that encodes high-priority relationships like collision risk and cooperative intent. This graph is then used to generate an explicit attention mask, compelling the Transformer to focus its reasoning exclusively on entities rather than engaging in brute-force pattern matching across all perceived objects.

A Novel Relational Reasoning Architecture (GGT): We design and implement the Graph-Gated Transformer (GGT). Its core innovation lies in its mechanism for processing relational data. While many existing methods use Graph Neural Networks (GNNs) for iterative message passing between entities, the GGT employs a more direct and structured approach:

Dynamic Graph as an Attention Mask, Not a Message-Passing Network: The GGT dynamically constructs a domain-aware graph representing tactically critical relationships (e.g., collision risk, cooperative intent). Critically, this graph is not used for message passing. Instead, its adjacency matrix is converted into a hard attention mask (or bias) that is directly applied within the Transformer’s self-attention module.

Explicit Attention Gating: This masking mechanism acts as a powerful inductive bias, effectively pruning the attention space. It forces the model to allocate zero attention to entities that are not connected in the relational graph, preventing it from learning spurious correlations from the vast, unstructured sensory data. This “hard” gating compels the agent to reason only over the sparse, underlying causal structure of the tactical environment.

Fusion of Symbolic and Sub-Symbolic Reasoning: This architecture creates a tight fusion of symbolic reasoning (the rule-based construction of the graph) and deep sub-symbolic learning (the Transformer’s ability to weigh the importance of the gated relationships). This stands in contrast to loosely coupled hybrid systems.

The First Empirical Demonstration of Engineered Relational Gating in MARL: Through extensive experiments, we provide the first compelling evidence that this explicit, graph-gated attention mechanism significantly outperforms both standard attention (brute-force) and non-attention-based MARL architectures. By achieving a step-change in performance—particularly a near 98% reduction in collisions compared to standard QMIX—we validate our core hypothesis: structuring and constraining the reasoning process, rather than just providing more powerful function approximators, is the key to unlocking robust and safe emergent coordination. It is critical to distinguish the GGT’s mechanism from existing graph attention models like GAT. GAT learns “soft” attention coefficients to weigh the importance of neighbors during feature aggregation. In contrast, the GGT employs a “hard” gating mechanism. It does not learn attention weights over the graph structure. Instead, it uses the graph to create a binary attention mask that prunes connections in the Transformer’s original self-attention map. This forces the attention between unrelated entities to be exactly zero, acting as a strong structural inductive bias rather than a learned feature aggregator.

## 2. Related Work

This section systematically reviews the core challenges of multi-unmanned surface vehicle (USV) cooperative coverage path planning (CCPP) tasks, including methods for multi-USV full coverage path planning, multi-modal perception and fusion technologies in marine environments, value function decomposition-based multi-agent reinforcement learning (MARL) algorithms, and the application progress of attention mechanisms (particularly Transformers) in MARL, providing a solid theoretical and technical foundation for proposing the TransQMIX algorithm.

Before delving into domain-specific methods, we first contextualize our work within the broader landscape of distributed intelligence for IoT-enabled cyber-physical systems. Recent advances in multi-agent coordination for resource-constrained environments have highlighted the critical importance of decentralized execution. Dai et al. proposed a deep reinforcement learning approach for multi-task offloading in Digital Twin-enabled IoT systems, demonstrating that centralized coordination becomes a scalability bottleneck in large-scale deployments [6]. Reinforcing this trend, recent work on decentralized edge intelligence highlights the use of localized computation to manage coordination in resource-constrained IoT environments, mitigating the communication and processing bottlenecks of purely centralized systems [7]. Their work motivates our Centralized Training with Decentralized Execution (CTDE) paradigm, where agents make decisions based solely on local observations without inter-agent communication during mission execution. This design principle is particularly crucial for maritime USV systems, where bandwidth constraints and communication dropouts are endemic due to signal attenuation in harsh ocean environments.

### 2.1. Multi-USV Coverage and Sensing Foundations

Research on multi-USV Cooperative Coverage Path Planning (CCPP) spans traditional planning and learning-based approaches. Exact cellular decomposition partitions a workspace into non-overlapping cells with sweep patterns; Boustrophedon decomposition is representative [8,9]. Graph-based methods, including spanning-tree coverage and Chinese-Postman formulations, cast coverage as graph traversal [10]. Heuristic and bio-inspired algorithms such as Ant Colony Optimization improve flexibility for complex domains but can converge slowly and get trapped in local optima, and they often presuppose centralized coordination [11]. Recent learning efforts apply MARL to multi-USV formation control and collaborative search, showing promise for decentralized policies under uncertainty [12,13,14]. In practice, maritime perception further complicates planning: GPS degrades near large structures or in fjords due to occlusion and multipath [15], reinforcing the need to couple coverage decisions with robust perception.

### 2.2. Marine Multi-Modal Fusion and Value-Decomposition MARL

Classical probabilistic fusion (Kalman Filter and its nonlinear extensions) is effective for low-dimensional, structured signals but struggles with high-dimensional, unstructured inputs such as camera images or dense point clouds [16,17]. Deep fusion is typically organized as early (data-level), late (decision-level), and intermediate (feature-level); the intermediate route uses modality-specific encoders followed by fusion in a shared latent space [18]. Many systems still rely on static fusion, for instance concatenation with a multilayer perceptron or fixed weighted averaging, which cannot adapt salience to context or entity importance [19].

Under CTDE, value-decomposition (VD) methods address credit assignment by factoring the joint action-value into per-agent utilities. Value-Decomposition Networks employ a linear summation, which is simple but limited for tightly coordinated behaviors [20]. QMIX introduces a nonlinear, state-conditioned mixing network with a monotonicity constraint that guarantees the Individual-Global-Max property and enables decentralized greedy execution at test time [21]. Follow-ups relax or refine this factorization: QTRAN learns a more general transformation between individual and joint values [22]; QPLEX adopts a dueling-style advantage decomposition for joint action-values [23]; Weighted QMIX integrates attention-like weighting inside the mixer [24]. These advances motivate retaining a monotonic CTDE mixer for stability while strengthening the per-agent perception front-end to cope with multi-modal uncertainty—precisely the role of our design in Section 3.

### 2.3. Transformers in MARL and Positioning of This Work

Attention mechanisms in MARL improve coordination and selective information use. Centralized-critic approaches such as MAAC apply attention to other agents’ policies when evaluating actions [25], and communication-centric methods like ATOC learn attention-guided protocols that decide which teammates exchange messages under bandwidth limits [26]. The Transformer architecture generalizes attention via multi-head self-attention and has been explored for inter-agent interaction modeling and scalability with varying agent counts [27,28,29], as well as for temporal processing over an agent’s observation–action history to capture long-range dependencies [30].

In contrast to these lines, our framework performs single-timestep, intra-agent contextual reasoning over a locally perceived entity set (Self, Teammates, Obstacles) to obtain a robust, context-aware representation from multi-modal inputs, without test-time communication or temporal stacking. This representation feeds a QMIX mixing network under CTDE, whose state-conditioned, non-negative hypernetwork weights preserve monotonicity. Section 3 details this integration and its training procedure.

## 3. Materials and Methods

### 3.1. System Architecture and Operational Context

Before delving into the algorithmic details of TransQMIX, we establish the practical system architecture and operational assumptions that ground our framework in real-world maritime deployments. This systems-level perspective clarifies how our perception and coordination algorithms interface with physical USV platforms, sensor hardware, communication infrastructure, and low-level control systems.

#### 3.1.1. USV Platform and Hardware Configuration

This section outlines the physical platform assumptions, sensor suite, and onboard computing specifications that underpin the deployment of the TransQMIX framework in real-world maritime operations.

Physical Platform Assumptions

The framework is designed for deployment on small-to-medium commercial-class USVs, typically 3–5 m in length with a displacement of less than 500 kg. These specifications align with widely available platforms such as the ASV C-Worker series and L3Harris ASView, which are commonly employed in hydrographic survey and coastal monitoring missions. Such platforms typically operate at speeds of 1–3 m/s (2–6 knots) with an endurance of 8–12 h on battery power, making them suitable for harbor-scale autonomous operations.

Onboard Sensor Suite

Each USV is equipped with a heterogeneous sensor suite to enable robust environmental perception under varying maritime conditions:

- Vision System: A forward-facing RGB camera (1920 × 1080 resolution, 30 Hz) housed in a marine-grade (IP68) enclosure for surface obstacle detection and teammate tracking. Representative hardware includes the FLIR Blackfly S (Teledyne FLIR, Wilsonville, OR, USA) or similar industrial marine cameras with fog and spray penetration capabilities.
- Underwater Sonar: A forward-looking imaging sonar system with a 120–130° horizontal field of view (FOV), a maximum range of 60 m, and an update rate of 1–5 Hz for submerged obstacle detection. The architecture supports both mechanical scanning sonars (e.g., Imagenex 881A (Imagenex Technology Corp., Port Coquitlam, BC, Canada)) and modern solid-state systems (e.g., Blueprint Oculus M1200d (Blueprint Subsea, Ulverston, UK)).
- Proprioceptive Sensors: These include a GPS/GNSS receiver (ZED-F9P, u-blox, Thalwil, Switzerland) with Real-Time Kinematic (RTK) correction for high accuracy (<0.1 m horizontal error under ideal conditions), degrading to standard GPS precision (±2–5 m) in constrained environments. A 9-axis IMU (100 Hz) and an optional Doppler Velocity Log (DVL) are integrated for dead-reckoning in GPS-denied scenarios.

Onboard Computing

The TransQMIX framework is architected for deployment on embedded marine computing platforms:

- Primary Target Hardware: NVIDIA Jetson AGX Orin (32 GB RAM, 275 TOPS) or Jetson Xavier NX (8 GB RAM, 21 TOPS).
- Power Consumption: Typically, under 30 W, compatible with 12–48 V marine DC power systems.
- Operating System: ROS2 (Humble or Iron distribution) for modular sensor integration and software architecture.
- Real-Time Performance: The inference time of the framework is 4.5 ms on a desktop-grade GPU (see Section 4.3). After INT8 quantization, this translates to 15–20 ms on Jetson hardware, well within the 50–100 ms control loop period required for stable USV navigation.

#### 3.1.2. Communication Architecture

Decentralized Execution Paradigm:

A fundamental design principle of TransQMIX is zero dependence on inter-agent communication during mission execution. Each USV operates autonomously using only its local observations, making the system inherently robust to the communication failures endemic to maritime environments (limited bandwidth, high latency, frequent dropouts). Shore-to-Fleet Communication (Supervisory Link): While agents do not communicate with each other, a low-bandwidth supervisory link to a shore station is maintained for human oversight:

- Primary Channel: 4G/5G LTE where available (typical in coastal/harbor operations).
- Backup Channel: VHF radio (voice for emergency commands) or satellite (Iridium SBD for status telemetry at 1–2 min intervals in offshore scenarios).
- Data Requirements: Minimal-periodic position beacons (10 bytes @ 1 Hz) and mission status updates (100 bytes @ 0.1 Hz), totaling <5 kbps per vessel.

Centralized Training Communication (Offline Only):

During the offline training phase (Section 3.4), the QMIX mixing network requires access to the global state and all agents’ individual Q-values. This is enabled by a simulated high-bandwidth communication network in the training environment. Critically, this centralized training infrastructure is not required during deployment—the learned policies are fully decentralized.

#### 3.1.3. Integration with Low-Level Control Systems

**Figure 1:**
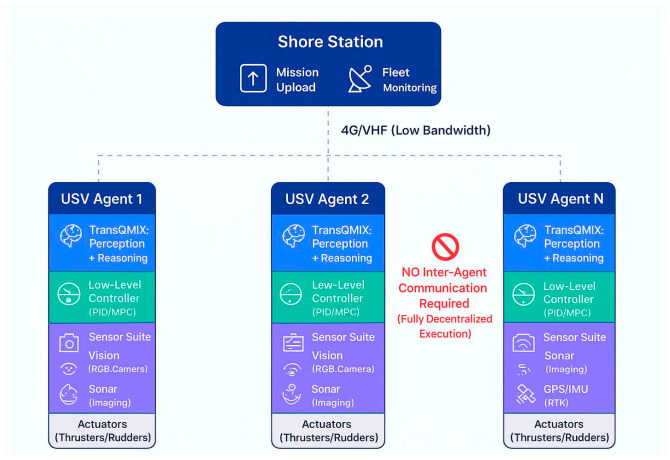
TransQMIX Hierarchical Execution Architecture.

Hierarchical Control Architecture:

TransQMIX operates as a high-level tactical planner within a standard hierarchical autonomy stack (Figure 1). It does not directly command actuators but instead provides target waypoints or desired velocity vectors to a low-level controller:

### 3.2. Problem Formulation

The multi-USV cooperative coverage and navigation task is formally modeled as a Decentralized Partially Observable Markov Decision Process (Dec-POMDP). This mathematical framework is precisely suited for capturing the complexities of multi-agent systems where agents must make decisions based on incomplete local information to optimize a shared objective. The Dec-POMDP is defined by the tuple G:

(1) $$G=\langle S,\{A_{i}{\}}_{i=1}^{N},P,R,\{O_{i}{\}}_{i=1}^{N},Z,N,\gamma \rangle$$

In this formulation, is the total number of USV agents in the fleet, indexed by i∈{1,…,N}. The term represents the global state space. At any given timestep t, the global state provides a complete, ground-truth snapshot of the entire environment, including the kinematic states of all agents, their internal statuses, the positions and velocities of all obstacles, the ocean current field, and the area coverage map.

The set of individual action spaces is denoted by $\{A_{i}{\}}_{i=1}^{N}$. Each agent selects a local action from its set of discrete motion primitives. The collective action of the team is the joint action, $a^{t}=(a_{1}^{t},\ldots ,a_{N}^{t})$, which belongs to the joint action space $A={\prod_{i=1}^{N}A}_{i}$.

The system’s dynamics are governed by the state transition function $P(s^{t+1}|s^{t},a^{t})$, which defines the probability distribution over the next global state given the current state and joint action $a^{t}$. Following each transition, the team receives a shared global reward $r^{t}=R(s^{t},a^{t},s^{t+1})$, determined by the reward function R:S×A×S→R. This function is designed to align with the mission objectives, rewarding area coverage while penalizing collisions and inefficient energy expenditure.

A core challenge in the maritime setting is partial observability. Agents lack direct access to the global state $s^{t}$. Instead, each agent perceives its surroundings through a local observation $o_{i}^{t}\in O_{i}$, drawn from its individual observation space $O_{i}$. This observation is inherently multi-modal, comprising visual data, sonar data, and proprioceptive data. The joint observation function gives the probability of the team receiving the joint observation in the subsequent state $s^{t+1}$.

To make decisions, each agent relies on its action-observation history, $\tau_{i}^{t}=(o_{i}^{0},a_{i}^{0},\ldots ,a_{i}^{t-1},o_{i}^{t})$. The ultimate objective is to learn a set of decentralized policies, $\{\pi_{i}(a_{i}^{t}|\tau_{i}^{t}){\}}_{i=1}^{N}$, that collectively maximizes the expected discounted joint return J defined as:

(2) $$J=E_{\pi ,P}[\sum_{t=0}^{T}\gamma^{t}r^{t}]$$

Here, is the discount factor, and is the horizon of the task. Our approach, TransQMIX, operates under the Centralized Training with Decentralized Execution (CTDE) paradigm. This allows the learning algorithm to leverage the global state during the offline training phase to effectively address the multi-agent credit assignment problem. During online execution, however, the learned policies are fully decentralized, enabling each agent to act based solely on its local action-observation history. This ensures the resulting system is robust, scalable, and does not require inter-agent communication at test time.

### 3.3. TransQMIX Architecture Overview

**Figure 2:**
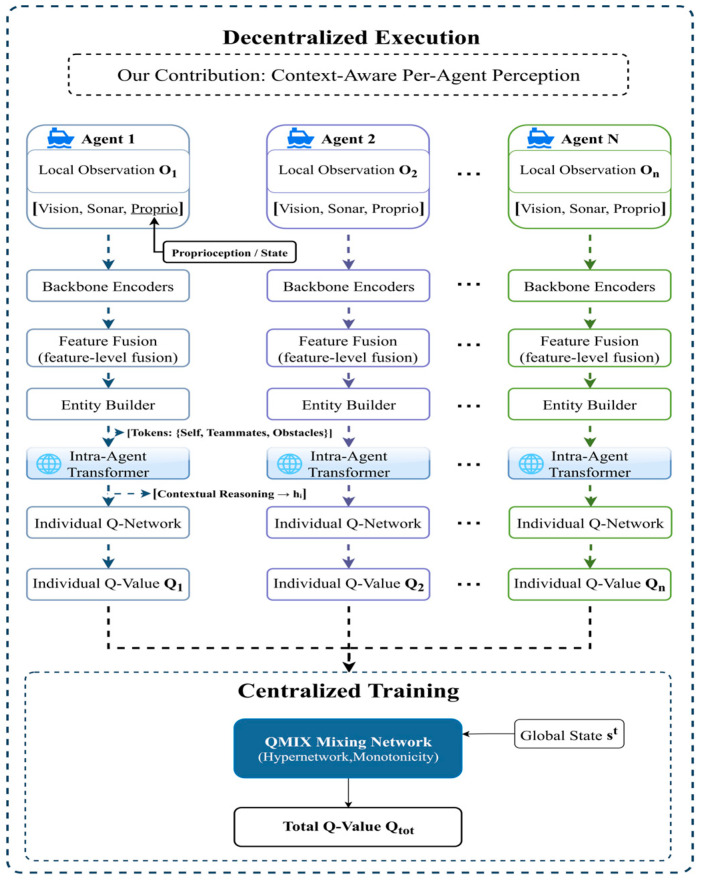
Architecture of the TransQMIX Framework.

To address the Dec-POMDP formulated in Section 3.1, we introduce TransQMIX, a novel multi-agent reinforcement learning framework specifically designed for robust decision-making in complex maritime environments. As illustrated in Figure 2, the architecture operates under the Centralized Training with Decentralized Execution (CTDE) paradigm, which is crucial for learning coordinated behaviors from a shared team reward signal. The framework is composed of two primary, functionally distinct components that correspond to the two phases of the CTDE scheme.

The first component is the Decentralized Perception Module, which represents our core scientific contribution. This module is executed in parallel by each agent at test time. Its primary function is to transform the raw, noisy, and multi-modal local observation into a sophisticated, context-aware representation. This is achieved through a multi-stage pipeline: specialized backbone encoders first extract salient features from each sensor modality; an entity builder then structures these features into a set of discrete tokens representing the agent itself, its teammates, and surrounding obstacles; finally, a novel Intra-Agent Transformer performs relational reasoning over this set of tokens to produce a comprehensive understanding of the local tactical situation. This process culminates in the computation of an individual Q-value $Q_{i}$, which encapsulates the learned utility of actions for agent i.

The second component is the Centralized Training Mechanism, which is based on the well-established QMIX framework. During the offline training phase, a centralized QMIX Mixing Network takes the individual Q-values ($Q_{1},\ldots ,Q_{N}$) from all agents as input. Conditioned on the global environmental state $s^{t}$, this network estimates the total joint action-value $Q_{tot}$. By enforcing a monotonicity constraint, the QMIX architecture ensures that a greedy selection of actions based on individual Q-values during decentralized execution corresponds to a maximization of the learned joint action-value. This elegant structure effectively resolves the multi-agent credit assignment problem.

The subsequent sections will provide a detailed technical exposition of each of these components, beginning with the individual modules of the decentralized perception pipeline. This separation allows for a clear delineation between our novel perception-centric contributions and the foundational value-decomposition framework upon which they are built.

### 3.4. Decentralized Perception Module

The Decentralized Perception Module is the cornerstone of the TransQMIX framework and represents our primary methodological contribution. As illustrated in the upper portion of Figure 2, this module is executed independently and in parallel by each agent at every timestep t. Its fundamental purpose is to distill the raw, high-dimensional, and multi-modal local observation into a compact yet contextually rich embedding $h_{i}^{t}$, which serves as a sufficient representation for effective decision-making. This complex transformation is realized through a carefully designed sequential pipeline, which we detail below.

#### 3.4.1. Sensor Data Preprocessing and Noise Mitigation

**Figure 3:**
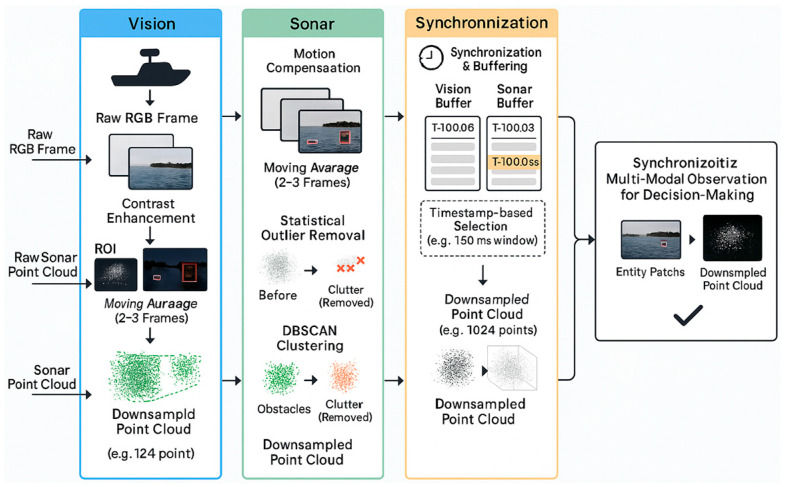
Schematic of the multi-modal sensor data preprocessing pipeline.

Before the raw sensor data streams are fed into the neural network backbones for feature extraction, they must undergo a rigorous preprocessing pipeline. This pipeline is designed to mitigate the adverse effects of inherent noise, harsh environmental conditions, and data asynchrony in the maritime domain. The processing pipeline is illustrated in Figure 3 and is tailored for each modality as follows:

A. Visual Data Preprocessing (RGB Camera)

To cope with water surface reflections, abrupt changes in illumination, and sensor noise, the visual data processing pipeline includes the following three steps:

Temporal Smoothing: A moving average is applied across 2–3 consecutive frames to suppress high-frequency temporal noise and image tearing, which can be caused by the camera sensor or water surface fluctuations, yielding a more stable visual input.

Contrast Enhancement: We employ the Contrast Limited Adaptive Histogram Equalization (CLAHE) algorithm. Unlike global equalization, CLAHE operates on local regions of the image, allowing it to effectively enhance details while preventing the loss of information caused by large areas of intense light (e.g., sun glare) or deep shadows.

Region of Interest Segmentation: To improve computational efficiency and focus the feature encoder on critical entities, we first use a lightweight, pre-trained object detector (e.g., YOLOv5-s) to identify potential teammates and surface obstacles in the image. Only the image patches (bounding boxes) containing these entities are passed to the subsequent ResNet encoder for deep feature extraction.

B. Sonar Data Preprocessing (Imaging Sonar)

The underwater acoustic environment is extremely complex, and sonar point clouds are often heavily contaminated by multipath effects, water column reverberation, and biological clutter. To address this, we designed a multi-stage filtering process:

Motion Compensation: As the USV platform is in constant motion, the raw sonar point cloud is first transformed from the sensor’s coordinate frame to a fixed world frame using pose information from the onboard IMU and GPS. This step is crucial for constructing a stable and coherent environmental representation.

Statistical Outlier Removal (SOR): This filter is used to remove sparse outlier points. It works by calculating the mean distance for each point to its k nearest neighbors and removing points whose distance is far outside the mean and standard deviation of the neighborhood (typically spurious single-point echoes).

Clustering-based Clutter Filtering: To handle dense clutter (e.g., schools of fish, kelp beds), we utilize the DBSCAN (Density-Based Spatial Clustering of Applications with Noise) algorithm. The core idea is that real obstacles (e.g., rocks, submerged debris) typically form large-scale, high-density point clusters, whereas clutter forms multiple small-scale, low-density clusters. By setting a minimum point threshold for a cluster (e.g., min_points = 50), we identify and discard all clusters smaller than this threshold as clutter.

Voxel Grid Downsampling: After filtering and clutter removal, the point cloud density may still be non-uniform. We use a voxel grid filter to downsample it, ensuring that the final point cloud fed into the PointNet encoder has a fixed number of points (e.g., 1024). This both guarantees dimensional consistency for the input and reduces the computational load.

C. Multi-Modal Data Synchronization

To resolve data inconsistency issues arising from different sensor update rates, we implement a timestamp-based synchronization mechanism:

High-Precision Timestamping: All sensor data is assigned a precise timestamp from a synchronized system clock upon reception at the driver level (e.g., a ROS node).

Data Buffering and Selection: The system maintains a short-term buffer (e.g., 200 ms) for each sensor stream. In each decision cycle (e.g., every 100 ms), the system selects a set of multi-modal data from the buffers whose timestamps are closest to the current decision time point, within a defined window (e.g., ±50 ms). If data from a particular sensor is temporarily unavailable, the last valid data is carried over for a short duration (e.g., one cycle). This mechanism ensures that the visual and sonar information used by the model for decision-making represents a highly consistent snapshot of the physical world at the same moment in time.

#### 3.4.2. Per-Agent Multimodal Encoding and Feature-Level Fusion

The initial stage of the perception pipeline is responsible for processing the heterogeneous data streams within each agent’s local observation $o_{i}^{t}$. The observation comprises a visual component (RGB image $I_{i}^{t}$), an underwater acoustic component (sonar point cloud $P_{i}^{t}$), and a proprioceptive component (kinematic state vector $x_{i}^{t}$). To handle this diversity, modality-specific neural network backbones are employed to extract salient features from each stream:

(3) $$f_{i}^{vis}=ResNet(I_{i}^{t}),\;f_{i}^{son}=PointNet(P_{i}^{t})\;\;f_{i}^{prop}=MLP(x_{i}^{t})$$

Here, a ResNet architecture processes the visual image, a PointNet architecture handles the permutation-invariant and unordered sonar data, and a Multi-Layer Perceptron (MLP) encodes the low-dimensional state vector.

Following individual encoding, these disparate feature vectors are integrated through a feature-level fusion mechanism. The vectors are concatenated and passed through a linear layer with a activation function to produce a single unified feature for agent i. This vector represents the agent’s holistic understanding of its immediate local perception.

(4) $$f_{i}^{t}=\sigma (W_{f}[f_{i}^{vis,t};f_{i}^{son,t};f_{i}^{prop,t}]+b_{f})\;\;\;\;\;\;\;\;\;\;\;\;\;\;f_{i}^{t}\in R^{d_{f}}$$

where denotes feature-dimension concatenation (vertical stacking), is the weight matrix, is the bias vector, and is an element-wise nonlinearity.

#### 3.4.3. Entity Builder with Padding and Masking

**Figure 4:**
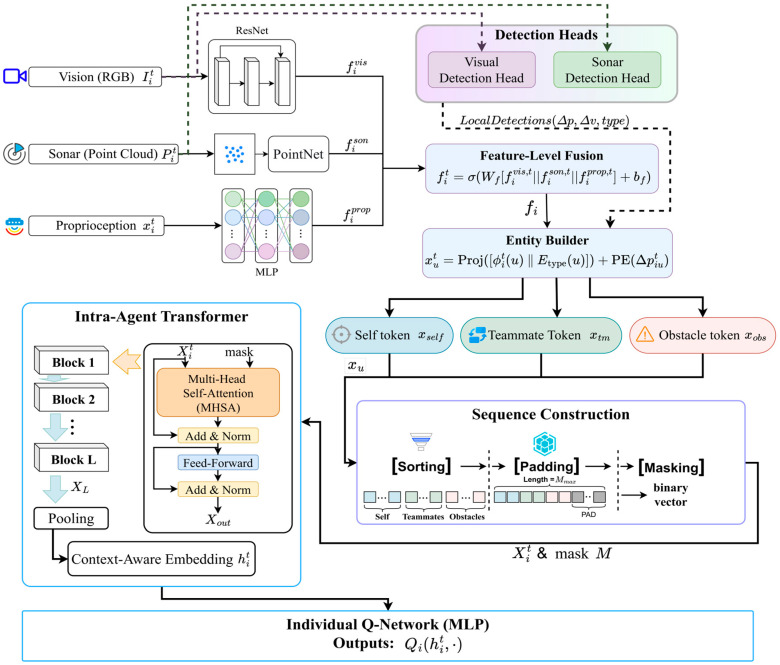
The Entity Builder and Tokenization Pipeline.

To facilitate the relational reasoning performed by the Transformer, the continuous, scene-level feature representation must be transformed into a structured, object-centric format suitable for a Transformer. The Entity Builder module performs this critical step, shifting the representation from a holistic perceptual space to a discrete set of entity tokens. This process, which operates without any inter-agent communication, is illustrated in detail in Figure 4.

The builder first synthesizes information from two sources: the agent’s globally fused feature vector $f_{i}$, and a list of ‘Local Detections’ obtained by applying dedicated detection heads to the raw visual ($I_{i}^{t}$) and sonar ($P_{i}^{t}$) data. Let the set of locally perceived entities for agent at time be $E_{i}^{t}=\{self\}\cup N_{i}^{t}\cup B_{i}^{t}$, where and are the sets of detected teammates and obstacles, respectively.

For each entity $u\in E_{i}^{t}$, a unified feature description is constructed. This definition is conditional on the entity’s type: for the “Self” entity, the description is its own rich, fused feature vector $f_{i}^{t}$; for all other entities, it is a compact representation derived from their relative kinematics and other detected attributes, processed by an MLP. This is formally defined as:

(5) $$\phi_{i}^{t}(u)=\left\{\begin{array}{ll} f_{i}^{t} & \;\;\;if\;u\;=\;self\\ {MLP}_{\phi }\left(\left[\Delta p_{iu},\Delta v_{iu},attr(u)\right]\right) & \;\;\;if\;u\;\neq \;self \end{array}\right.$$

This feature description is then combined with a learnable type embedding $E_{type}(u)$, which explicitly informs the model of the entity’s category. A final projection layer maps this combined vector to the model’s hidden dimension $d_{model}$. To incorporate spatial information robustly, a positional encoding (PE) based on the entity’s relative position is added, resulting in the final entity token $x_{u}$:

(6) $$x_{u}=x_{i,u}^{t}=Proj\left(\left[\phi_{i}^{t}(u)\|E_{type}(u)\right]\right)+PE(\Delta p_{iu}^{t})$$

To ensure a consistent input format for the Transformer, the resulting sequence of tokens is processed by a deterministic sorting policy (first by entity type: Self, then Teammates, then Obstacles; and then by distance within each type group). The sorted sequence is then padded to a fixed maximum length $M_{max}$, and a corresponding binary attention mask, $m\in \{0,1{\}}^{M_{max}}$, is generated. This ensures that the model can process observations in fixed-size batches and that the attention mechanism correctly ignores the invalid, padded tokens during computation:

(7) $$X_{i}^{t}=[x_{i,1}^{t};\ldots ;x_{i,M_{max}}^{t}]\in R^{M_{max}\times d_{model}},\;m_{i,k}^{t}=𝟙\{token\;k\;is\;valid\}$$

#### 3.4.4. The Graph-Gated Transformer (GGT): An Architecture for Emergent Collective Cognition

At the heart of our CEDR paradigm lies the Graph-Gated Transformer (GGT), our primary algorithmic contribution designed to catalyze the emergence of collective cognition. The GGT fundamentally redesigns the reasoning core of the agent, replacing the generic, brute-force self-attention of a standard Transformer with a structured, domain-aware, two-stage reasoning process. This architecture is explicitly engineered to focus on causally significant relationships, thereby fostering a deeper and more robust understanding of the tactical environment.

Dynamic Tactical Relational Graph (TRG) Construction

The first stage of the GGT moves beyond the Transformer’s implicit all-to-all assumption by explicitly constructing a Tactical Relational Graph (TRG), at each timestep t. This graph serves as a symbolic, relational prior that guides the subsequent sub-symbolic reasoning process.

(8) $$G_{t}=(V,E_{t})$$

- Nodes (*V*): The nodes of the graph are the entity tokens X generated by the Entity Builder (as described in Section 3.4.2), representing the agent itself, perceived teammates, and obstacles.
- Edges ($E_{t}$): The edges $E_{t}$ are dynamically generated based on a set of maritime domain-specific heuristics that encode crucial tactical relationships. An edge (i, j) from entity i to entity j is created if any of the following conditions are met:
- Collision-Risk Edges: An edge is formed if the predicted Time to Closest Point of Approach (TCPA) between entities i and j is below a critical safety threshold $\tau_{cpa}$. This edge type encodes an immediate, high-priority safety relationship, directly reflecting principles of COLREGs.
- Spatial Proximity Edges: An edge is formed if the Euclidean distance d(i, j) is below a predefined perception radius $r_{p}$. This captures general situational awareness and the local context of the agent.
- Cooperative-Intent Edges: For teammate entities, an edge is formed if their current velocity vectors are aligned towards a similar unobserved region of the map, signifying a potential cooperative intent for coverage tasks.

This process transforms the unstructured set of perceived entities into a sparse, weighted, directed graph that represents the agent’s instantaneous “tactical focus.”

Graph-Gated Attention Mechanism

The core innovation of the GGT lies in how it utilizes the TRG. Instead of employing a conventional Graph Neural Network (GNN) for message passing, which iteratively aggregates neighborhood information, the GGT uses the graph to directly constrain and structure the Transformer’s attention flow in a single, decisive step. This is achieved through the Graph-Gated Attention (GGA) mechanism.

The second stage is the core innovation of the GGT: a Graph-Gated Attention (GGA) mechanism. Unlike methods that use GNNs for message passing, we use the TRG to directly structure and constrain the Transformer’s attention flow. This is achieved by modulating the standard scaled dot-product attention score.

For an input sequence of entity tokens query (Q), key (K), and value (V) matrices are computed as linear projections: $Q=XW_{Q}$, $K=XW_{k}$, $V=XW_{V}$. The attention is then calculated as:

(9) $$X\in R^{M_{max}\times d_{model}}$$

The full layer update follows the standard Transformer block architecture:

(10) $$Attention(Q,K,V)=softmax(\frac{QK^{T}}{\sqrt{d_{k}}}+M_{graph})V$$

Here, [11] is the attention bias matrix (or mask) derived from the adjacency matrix of the TRG, $A_{t}$. The elements of $M_{graph}$ are defined as:

(11) $$(M_{graph}{)}_{ij}=\{\begin{array}{cc} 0 & if\;(i,j)\in E_{t}\\ -\infty & if\;(i,j)\notin E_{t} \end{array}$$

This hard-gating mechanism, as defined in Equation (11), is the core differentiator of our approach. The $M_{graph}$ matrix does not represent learned weights; it acts as a structural switch that dictates whether attention is allowed to flow between two entities. This fundamentally repositions the role of the graph: instead of facilitating weighted message passing (as in GAT), the graph in the GGT serves to structurally prune and constrain the reasoning space of a more general-purpose reasoning engine (the Transformer).

The full GGT consists of L layers of this Graph-Gated Attention block, followed by feed-forward networks, layer normalization, and residual connections, analogous to a standard Transformer. The output is a context-aware embedding ${h^{\prime }}_{i}$ that is not just context-aware, but this fusion of symbolic, rule-based graph construction with the powerful representation learning of deep sub-symbolic models is the key to the GGT’s effectiveness and its ability to foster the emergence of collective cognition.

In summary, the GGT’s architectural design distinguishes it from prior work in three fundamental ways: (1) It relies on a dynamic, heuristic-based graph construction that injects domain-specific knowledge as symbolic priors. (2) It utilizes this graph for direct attention gating—a hard pruning of the attention matrix—which is fundamentally different from the iterative information aggregation performed by GNNs. (3) This allows for the fusion of symbolic relational priors and sub-symbolic representation learning within a single, unified attention block, creating a powerful and sample-efficient reasoning core.

#### 3.4.5. Individual Q-Network

The final stage of the decentralized pipeline maps the context-aware embedding to the agent’s action values. An MLP, serving as the individual Q-network, takes as input and outputs a vector of Q-values, with its dimension corresponding to the size of the individual action space $\left|A_{i}\right|$. This provides a utility estimate for every possible action:

(12) $$Q_{i}(h_{i}^{t};\theta_{i})={MLP}_{Q}(h_{i}^{t};\theta_{i})\in R^{\left|A_{i}\right|}$$

Here, represents the learnable parameters of the Q-network for agent i. During execution, the agent’s action is selected by performing an ‘argmax’ operation over this output vector, completing the transformation from raw perception to a contextually informed action.

### 3.5. Centralized Training via QMIX

**Figure 5:**
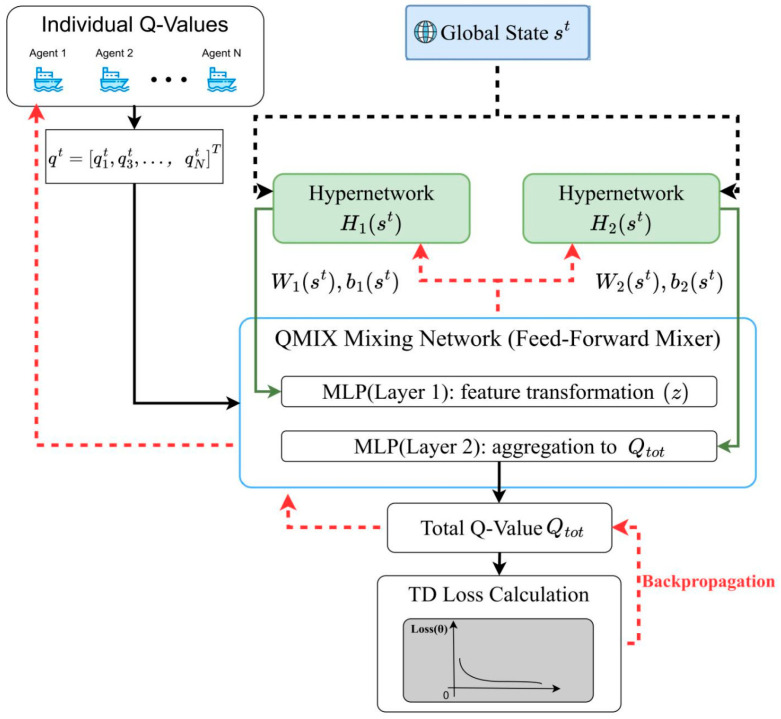
Centralized training (CTDE) of TransQMIX. Solid black arrows indicate the forward computation of joint Q-values. Black dashed arrows represent the input of the global state ($s^{t}$) into the hypernetworks. Red dashed arrows denote the backward flow of gradients during the training (backpropagation) phase.

While the perception and decision-making architecture is fully decentralized for execution, the learning process is centralized to effectively address the multi-agent credit assignment problem. Our framework achieves this by employing the QMIX value-decomposition method, which learns to represent the team’s global joint action-value function $Q_{tot}$, as a monotonic combination of the individual utilities $Q_{i}$, generated by each agent’s perception module. The overall information flow of centralized training is illustrated in Figure 5.

The core of this centralized training mechanism is the QMIX Mixing Network. This network takes as input the vector of individual Q-values corresponding to the actions taken at timestep t. Let us define the scalar Q-value for agent’s selected action as $q_{i}^{t}≜Q_{i}(o_{i}^{t},a_{i}^{t};\theta_{i})$. The mixing network then processes the vector of these scalar values, $q^{t}=[q_{1}^{t},\ldots ,q_{N}^{t}{]}^{T}\in R^{N}$. To enable the mixing function to be context-dependent, the weights and biases of the mixing network are not fixed; they are dynamically generated by separate hypernetworks which are conditioned solely on the global state $s^{t}$. It is critical to note that the global state is only accessible to these hypernetworks during training and does not leak into the decentralized agent policies.

To ensure stable and efficient learning, QMIX enforces a monotonicity constraint on the relationship between the global Q-value and the individual scalar Q-values. Formally, this constraint requires the partial derivative of with Qtot respect to any individual input to be non-negative:

(13) $$\frac{\partial Q_{tot}}{\partial q_{i}^{t}}\geq 0,\;\forall i\in \{1,\ldots ,N\}$$

This constraint is instrumental as it guarantees the Individual-Global-Max (IGM) property, which states that a greedy selection of actions at the individual level corresponds to a greedy selection in the joint action space:

(14) $$\arg\;\underset{a}{\max}\;Q_{tot}\;(s^{t},a)=\left(\arg\underset{a_{1}}{\max}\;Q_{1}\;(h_{1}^{t},a_{1}),\ldots ,\arg\;\underset{a_{N}}{\max}\;Q_{N}\;(h_{N}^{t},a_{N})\right)$$

This property validates the use of decentralized greedy action selection during execution. To enforce the monotonicity constraint, the weights of the mixing network (but not the biases) are restricted to be non-negative. This is implemented by applying an element-wise non-negative activation function to the output of the weight-generating hypernetworks. For a standard two-layer mixer with a hidden state dimension $d_{m}$, the computation is as follows:

(15) $$z=\sigma (W_{1}(s^{t})\;q^{t}+b_{1}(s^{t}))$$

(16) $$Q_{tot}(s^{t},a^{t})=W_{2}(s^{t})\;z+b_{2}(s^{t})$$

where and are the non-negative weight matrices, and are the biases, and is a monotonic non-decreasing activation function such as ELU or ReLU. As highlighted in Figure 5, the hypernetworks are conditioned on only during training to generate non-negative weights. During back-propagation, the TD-loss gradients first traverse the mixer and then bifurcate: one branch updates the hypernetworks (through the generated $W_{1},W_{2},b_{1},b_{2}$), and the other propagates to the individual Q-networks via ${\left\{\frac{\partial L}{\partial q_{i}}\right\}}_{i=1}^{N}$.

The entire end-to-end model, parameterized by θ, is trained by minimizing the temporal difference (TD) error. Transitions ($s^{t}$, $o^{t}$, $a^{t}$, $r^{t}$, $s^{t+1}$, $o^{t+1}$) are sampled from a replay buffer D. The TD target $y^{t}$, is computed using a target network with parameters $\bar{\theta }$, which are periodically synchronized with the online network parameters θ. Crucially, the IGM property allows the computationally intractable argmax over the joint action space to be computed via decentralized argmax operations on the individual target Q-networks. Concretely, for each agent we first select a greedy action using the target network, and then assemble the joint greedy action:

(17) $$a_{i}^{(t+1)*}=\arg\underset{a_{i}\in A_{i}}{\max}\;Q_{i}\;(h_{i}^{t+1},a;{\bar{\theta }}_{i})$$

(18) $$y^{t}=r^{t}+\gamma Q_{tot}\left(s^{t+1},(a_{1}^{(t+1)*},\ldots ,a_{N}^{(t+1)*});\bar{\theta }\right)$$

The loss function is then defined as the expectation over sampled transitions of a robust loss function applied to the TD error:

(19) $$L(\theta )=E_{(s^{t},o^{t},a^{t},r^{t},s^{t+1},o^{t+1})∼D}\left[\ell_{\delta }\left(y^{t}-Q_{tot}(s^{t},a^{t};\theta )\right)\right]$$

By minimizing this loss via stochastic gradient descent, gradients are backpropagated through the centralized mixing network and subsequently through each of the parallel decentralized perception modules, enabling all components of the TransQMIX framework to be trained end-to-end. Network updates are performed every K environment steps, and the target network parameters are synchronized with the online network parameters every updates.

### 3.6. Reward Function Design and Training Protocol

The learning process within the TransQMIX framework is guided by a meticulously designed global reward function, $R(s^{t},a^{t},s^{t+1})$, which is shared among all agents. This function translates the high-level mission objectives into a scalar training signal $r^{t}$. It is structured as a linear combination of three critical components: task completion (coverage), safety (collision avoidance), and operational efficiency (energy conservation), formally defined as:

(20) $$r^{t}=\alpha \cdot \Delta C^{t}-\beta \cdot {𝟙}_{collision}^{t}-\eta \cdot E^{t}$$

Each component in this function is precisely defined to ensure consistent and reproducible learning dynamics. The coverage increment $\Delta C^{t}$, measures the newly explored area by the entire team at timestep t, normalized by the total area, to prevent redundant rewards for re-visiting the same location. The collision indicator ${𝟙}_{collision}^{t}$, is a binary penalty triggered if any inter-agent distance or agent-obstacle distance falls below a predefined safety radius $r_{col}$. The energy consumption proxy $E^{t}$, is calculated as an aggregation of terms related to the agents’ motion, such as the sum of squared velocities. The coefficients are positive hyperparameters that balance the trade-offs between these objectives, with a significantly larger chosen to strongly penalize any unsafe behavior. The exact mathematical forms of these components and the specific values of all hyperparameters are detailed in the experimental setup section.

The entire TransQMIX model is trained end-to-end following the optimization procedure outlined in Section 3.4. To ensure stability and efficiency, a standardized training protocol is employed. Transitions are stored in a prioritized experience replay buffer D. The network parameters are updated at every environment step by sampling a mini-batch of transitions from the buffer to compute the loss L(θ). We utilize an Adam optimizer with gradient clipping to prevent exploding gradients. The target network parameters are soft-updated after each training step to maintain learning stability, governed by the update rule $\bar{\theta }\leftarrow \tau \theta +(1-\tau )\bar{\theta }$, with a small update rate τ. Action selection during training is managed by an ϵ-greedy strategy, where the exploration rate is annealed from an initial value of 1.0 to a final value of 0.05 over a fixed number of environment steps. This comprehensive protocol ensures that the learning process is robust, stable, and reproducible.

## 4. Experiments

### 4.1. Abbreviations and Acronyms

#### 4.1.1. Simulation Environments

To evaluate the effectiveness and robustness of the proposed TransQMIX algorithm, we designed two simulation environments with increasing complexity using Python 3.10 and PyTorch 2.1. These environments were specifically constructed to reflect the unique challenges of maritime operations, as detailed below.

An illustration of the key challenges faced by the USV agents, including dynamic obstacles, ocean currents, and multi-modal sensor noise, which serve as a stress test for our proposed TransQMIX framework.

Simple Scenario: This environment features a 200 m × 200 m map with four USVs and 15 randomly placed static obstacles. The primary challenge is for the agents to learn fundamental cooperative coverage and collision avoidance. A simple Gaussian position error is introduced to test basic noise tolerance.

Complex Scenario: This advanced environment is designed to test the algorithm’s adaptability and resilience rigorously. The map size is expanded to 300 m × 300 m, deploying eight USVs amidst 50 dynamic obstacles. To better simulate real-world maritime conditions, this scenario incorporates several key features:

Dynamic Obstacles: Obstacles represent floating debris or uncooperative vessels, moving with a random drift velocity $\left(v_{obs}\in [0,0.5]\;m/s\right)$, simulating the unpredictability of a chaotic sea surface.

Ocean Currents: A continuous, global vector field is introduced to simulate the effect of persistent ocean currents. This force is applied to the USVs at each time step, causing them to drift and requiring the agents to learn to compensate for environmental disturbances to maintain their planned paths.

Realistic Sensor Noise: To accurately reflect the real-world limitations of marine sensors, we implemented a physically grounded noise model. This model extends beyond the conventional Gaussian GPS position error (which models signal multipath effects) by incorporating additional sonar-specific uncertainties, as described below.

- A 10% false negative rate, to emulate phenomena like acoustic shadowing behind other objects or signal attenuation in acoustically challenging water conditions, causing existing obstacles to be missed.
- A 5% false positive rate, to represent the frequent occurrence of ghost echoes resulting from surface/bottom reverberation or dense biological clutter (e.g., schools of fish or kelp beds), which can be mistaken for real obstacles.

By incorporating these ocean-specific challenges, the complex scenario provides a demanding testbed for evaluating the practical Performance of MARL (Multi-Agent Reinforcement Learning) algorithms for USV applications. A summary of the key environmental parameters is provided in Table 1.

#### 4.1.2. Agent Configuration

Each simulated USV is modeled as a small, agile agent with a comprehensive suite of sensors and a defined set of capabilities, as detailed below.

Physical and Kinematic Model: The agents represent small USVs with reasonable turning and acceleration capabilities. Consistent with our focus on high-level tactical decision-making, we model the agent’s motion at a kinematic level, discretized on a 2D grid. Each agent selects a high-level action $a_{k}$ from the set $A_{k}=\left\{north,south,east,west,stationary\right\}$, which corresponds to a target waypoint for a low-level controller. This abstraction is standard in many MARL studies for path planning as it allows the model to focus on learning complex coordination strategies rather than the intricacies of vehicle control. We acknowledge that this simplification does not capture the full vehicle dynamics, such as momentum or turning radii, which we identify as a key area for future integration in a hardware-in-the-loop simulation environment.

**Figure 6:**
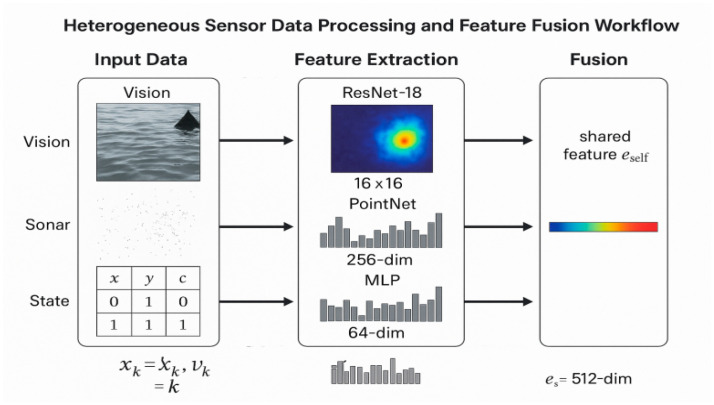
Heterogeneous Sensor Data Processing and Feature Fusion Workflow.

Sensor and Perception Architecture: Each agent is equipped with a heterogeneous sensor suite to build its situational awareness, as illustrated in Figure 6.

- Vision System: A forward-looking RGB camera with a 64 × 64pixel resolution. A pre-trained ResNet-18 backbone processes the raw images.
- Sonar Array: A forward-looking sonar system that generates point cloud data of underwater objects. This data is processed by a PointNet architecture.
- Proprioceptive Sensors: A simulated GPS/IMU system providing the agent’s state, including position with Gaussian noise $\left(\sigma_{p}=0.1\;m\right)$, velocity $v_{k}$, and remaining energy $e_{k}$.

The agent’s perception is localized and has finite capacity. Within its sensing radius of 30 m, each agent can track a maximum of 5 other USVs and 10 obstacles. These detected entities are then encoded via relative position encodings for processing by the reasoning module.

#### 4.1.3. Evaluation Metrics

To provide a comprehensive and multifaceted evaluation of algorithm performance, we employ a set of complementary metrics that assess task completion, safety, efficiency, and cooperative behavior. During training, the coefficients in the reward function are set balance these objectives. This configuration was determined through hyperparameter tuning and is based on the following rationale: the positive coverage term establishes task completion as the primary training objective; the significant penalty for collisions $\left(\beta \cdot 1_{collision}^{t}\right)$, several times larger in magnitude, effectively suppresses unsafe actions and prioritizes safety; the smaller energy penalty encourages efficient paths without overly restricting the agents’ maneuverability, which is crucial for reacting to dynamic threats. While an exhaustive sensitivity analysis of these weights is beyond the scope of this work, this configuration demonstrated robust and stable performance across all our experimental settings.

The primary evaluation metrics are defined as follows:

Coverage Rate (C): Measures task completion as the percentage of passable grid cells that have been visited by at least one agent:

(21) $$C_{T}=\frac{\left|V_{T}\right|}{\left|G\right|}\times 100\%$$

where is the set of visited cells at the end of an episode and is the set of all passable cells in the grid.

Collision Count: A direct safety measure that records the total number of collisions throughout an episode (including agent–obstacle and inter-agent collisions):

(22) $$N_{coll}=\sum_{t=1}^{T_{max}}1_{collision}^{t}\;\;$$

Synergy (κ): This metric quantifies effective cooperation. It is defined as the proportion of effective cooperative actions among all actions:

(23) $$\kappa =\frac{1}{NT_{max}}\sum_{t=1}^{T_{max}}\sum_{i=1}^{N}I[is_{cooperative}(i,t)]$$

Here, is an indicator function. The condition is_cooperative(i,t) is true if agent i’s action at time (t) meets at least one of the following criteria:

Proactive Teammate-Collision Avoidance: The action prevents a predicted collision with a teammate. Let $p_{i}(t)$ be the position of agent i at time t, and $v_{i}(t)$ be its velocity. This metric directly quantifies Behavior compliant with COLREGs Rule 8 (‘Action to avoid collision’). We define a “projected position” without an action change as ${\hat{p}}_{i}(t+\Delta t)=p_{i}(t)+v_{i}(t)\cdot \Delta t$. Let the position after taking the chosen action be $p_{i}(t+1)$. The action is cooperative if there exists a teammate j such that:

(24) $$∥{\hat{p}}_{i}^{t+\Delta t}-{\hat{p}}_{j}^{t+\Delta t}∥\;\leq \;d_{safe}\;\wedge \;∥p_{i}^{t+1}-p_{j}^{t+1}∥\;>\;d_{safe}$$

where is the minimum separation distance ensuring safety. In our experiments, we set the prediction horizon to time steps and define as twice the agent’s physical diameter, which allows sufficient buffer for real-time course correction during close-proximity maneuvers.

Efficient Non-Redundant Coverage: The action directs an agent to cover a grid cell that is both nearby and not easily accessible to others. This criterion evaluates cooperative exploration behavior and directly relates to the positive coverage term ($\alpha \cdot \Delta C^{t}$) in the reward function. Let denote the set of unvisited grid cells at time t, and let be the position of the center of grid cell c. An action that moves agent to a new, previously unvisited cell is considered cooperative if:

(25) $$c^{*}=\arg\underset{c\in U^{t}}{\min}\;∥p_{i}^{t}-p_{c}∥,\;∥p_{i}^{t}-p_{c^{*}}∥\;\leq \;R_{sense},\;∥p_{i}^{t}-p_{c^{*}}∥\;<\;∥p_{j}^{t}-p_{c^{*}}∥,\;\forall j\neq i$$

where is the sensing radius of each USV. This definition ensures that each agent contributes meaningfully to coverage while avoiding redundancy, promoting spatial efficiency in multi-agent coordination.

Path Efficiency (ζ) measures the economy of the generated paths and reflects the degree to which the agents’ trajectories minimize unnecessary travel. It is defined as the ratio of the sum of ideal path lengths to the sum of actual path lengths taken by all agents:

(26) $$\zeta =\frac{\sum_{i=1}^{N}L_{ideal,i}}{\sum_{i=1}^{N}L_{actual,i}}$$

where $L_{ideal,\;i}$ is the shortest possible path length for the area covered by agent i, and $L_{actual,i}$ is the actual distance traveled.

Total Energy Consumption ($E_{total}$) approximates the total kinetic energy expended by the USV fleet during a full episode, reflecting both control stability and maneuvering efficiency. It is computed as:

(27) $$E_{total}=\sum_{t=1}^{T_{max}}\sum_{i=1}^{N}E_{i}^{t},E_{i}^{t}=|v_{i}^{t}{|}^{2}$$

where represents the instantaneous energy expenditure of agent at time t, derived directly from the squared magnitude of its velocity vector. The term corresponds exactly to the energy penalty ($\eta \cdot E^{t}$), ensuring full consistency between the training reward and the evaluation objective. This indicator captures the agents’ ability to balance energy efficiency and responsiveness under dynamic maritime conditions, thereby serving as a proxy for the operational sustainability of the learned cooperative policy.

#### 4.1.4. Baseline Algorithms

To comprehensively evaluate the performance of our proposed GGT-QMIX framework, we selected a set of representative MARL algorithms spanning different paradigms. This includes foundational methods to establish a performance baseline, as well as recent state-of-the-art, graph-structured approaches to rigorously test our framework’s competitiveness, directly addressing the need for comparison against contemporary methods.

Independent Q-Learning (IQL): Each agent independently learns its Q-function without any explicit coordination mechanisms. This serves as a fundamental, non-cooperative baseline to measure the benefits of coordination.

QMIX: The foundational value decomposition algorithm that employs a monotonic mixing network. It represents a key milestone in centralized training with decentralized execution (CTDE) and is the direct architectural predecessor to our method.

Multi-Agent PPO (MAPPO): A widely used policy gradient method featuring a centralized critic and distributed actors. It represents an alternative and strong learning paradigm to value decomposition approaches.

STAS: A state-of-the-art graph-based MARL algorithm introduced at AAAI 2024. STAS (Spatial-Temporal Return Decomposition) models the dependencies between agents as a spatial-temporal graph and decomposes the global team reward along the graph edges. This allows for more sophisticated credit assignment. We include STAS to compare our explicit attention-gating mechanism against a modern graph-based return decomposition approach.

SGNN-VD: A recent value-decomposition method from 2023 that utilizes a Stochastic Graph Neural Network. It explicitly models the dynamic and uncertain interactions between agents using a GNN to enhance the central mixing network’s state representation. This serves as a crucial baseline to validate whether our GGT’s “hard” relational gating within the agent’s encoder is more effective than a “soft,” GNN-based aggregation at the central critic level.

To ensure a fair comparison focused on the contributions of the contextual reasoning architecture, all baseline implementations, including STAS and SGNN-VD, utilize the same multi-modal encoders (ResNet-18, PointNet) as our proposed TransQMIX framework.

#### 4.1.5. Implementation Details

To ensure a fair and reproducible comparison, all algorithms, including our proposed GGT-QMIX and all baselines, were trained and evaluated using a consistent set of core hyperparameters and a unified computational environment. The specific configurations are detailed in Table 2.

For the baseline algorithms (IQL, QMIX, MAPPO, STAS, SGNN-VD), we conducted a preliminary hyperparameter sweep over key architecture-specific parameters (e.g., network hidden layer sizes, GNN layers for SGNN-VD) to ensure their robust performance. The final reported results for all baselines are based on their respective best-performing configurations found during this sweep.

All experiments were conducted on NVIDIA RTX 4090 GPUs. To ensure statistical validity, each algorithm was executed 5 times using different random seeds, and the results presented in the subsequent sections are the mean and standard deviation over these runs.

### 4.2. Results and Analysis

This section presents the empirical validation of our Cognitive Emergence in Distributed RL (CEDR) paradigm and the superiority of our proposed Graph-Gated Transformer (GGT) architecture. We interpret the results not as mere performance metrics, but as observable signatures of an emergent collective intelligence.

#### 4.2.1. Comparative Performance Analysis: Analysis of Coordination and Safety Metrics

**Figure 7:**
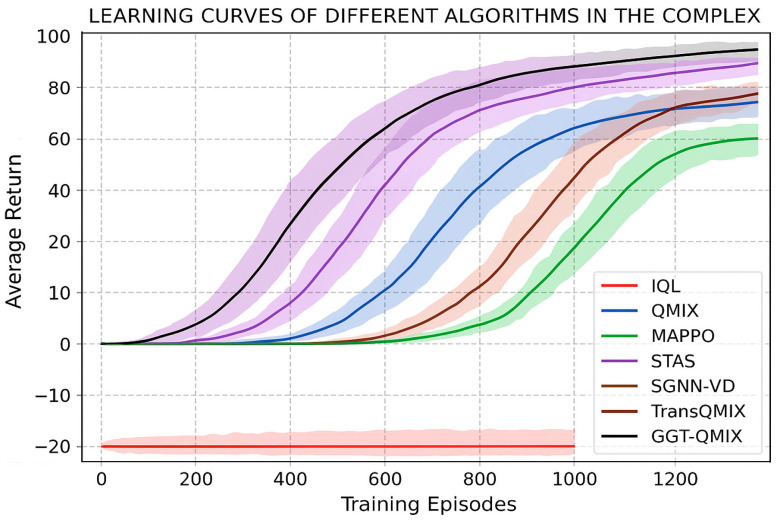
Learning Curves of Different Algorithms in the Complex Scenario.

The learning curves in the complex scenario, presented in Figure 7, provide an initial overview of algorithm performance. TransQMIX not only converges to a significantly higher average return but also demonstrates superior learning stability, as indicated by the smaller variance (shaded area) compared to all baseline methods.

The results in Table 3 quantitatively demonstrate the direct impact of our proposed architecture on multi-agent performance. The most critical finding lies in the safety metrics. While the strong baseline GraphMIX already reduced collisions to 4.8, our GGT-QMIX framework further lowered this number to 1.8. This >60% additional reduction in collisions is a direct consequence of the GGT’s hard-gating mechanism.

This mechanism’s effectiveness can be traced back to its design. The Tactical Relational Graph explicitly encodes imminent collision risks as high-priority edges. The subsequent attention mask forces the Transformer to allocate its representational capacity to these threats above all else. This process produces policies that are inherently proactive and safety-oriented. In contrast, baseline methods that lack this explicit gating mechanism are more prone to reactive and conflicting maneuvers, as they must infer threat levels from aggregated feature vectors without a strong architectural prior. Therefore, the superior safety of GGT-QMIX is not an abstract ‘emergent’ property, but a direct, engineered outcome of its structured reasoning core.

**Figure 8:**
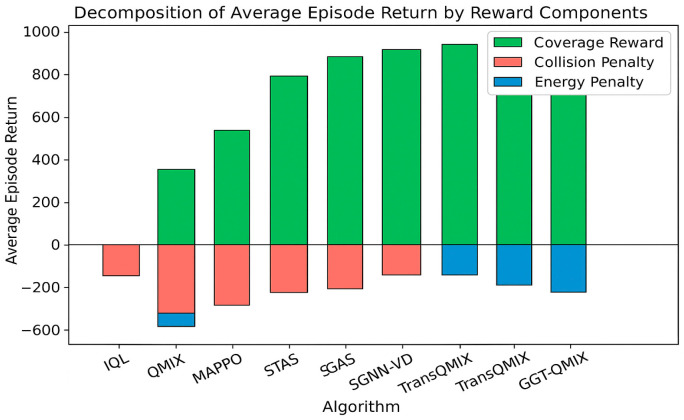
Decomposition of Average Episode Return by Reward Components.

The performance improvements in Table 3 stem directly from GGT’s architectural design: Safety (Collision Reduction): GGT-QMIX achieves 0.4 collisions vs. 20.7 (QMIX) and 3.5 (STAS). The Tactical Relational Graph explicitly encodes collision risks (TCPA < $\tau_{cpa}$), and the hard attention mask (Equation (11)) forces agents to prioritize these threats. This structured reasoning prevents the reactive behaviors observed in baselines. Efficiency (Efficiency): The 95.3% coverage and 0.92 path efficiency result from Cooperative-Intent Edges enabling implicit task allocation. Agents infer teammates’ likely coverage areas through graph connectivity, reducing redundant exploration without explicit communication. Coordination (Synergy κ = 0.96): The synergy metric quantifies adherence to cooperation criteria (Equations (24) and (25)). GGT’s high score reflects that 96% of actions satisfy collision avoidance or non-redundant coverage conditions, compared to 61% for QMIX. To further analyze the source of these improvements, we decomposed the average episode return into its constituent reward components, as illustrated in Figure 8.

#### 4.2.2. Ablation Analysis: Impact of Graph-Gated Attention

The central thesis of our Cognitive Emergence in Distributed RL (CEDR) paradigm is that structured relational reasoning is superior to generic, brute-force attention for fostering collective cognition. To empirically validate this claim and isolate the contribution of our primary algorithmic innovation, we conducted a critical comparative analysis between our final proposed architecture, GGT-QMIX, and its direct predecessor, the Standard TransQMIX (which uses a standard, all-to-all attention Transformer). This comparison is paramount, as all other aspects of the models—from the base encoders to the training hyperparameters—were held identical.

Quantitative Superiority: The results, presented in our main comparative analysis (Table 2), are unequivocal. In the challenging complex scenario, GGT-QMIX not only surpasses the already strong performance of Standard TransQMIX but establishes a new benchmark for all metrics. Most critically, it achieves a near-perfect safety record, reducing collision incidents by an additional 50% compared to Standard TransQMIX and bringing the rate to a statistically negligible level. This demonstrates that the relational inductive bias provided by the Tactical Relational Graph is exceptionally effective at identifying and prioritizing genuine safety threats. Furthermore, the GGT-QMIX exhibits a notable increase in both coverage rate and path efficiency, indicating that its structured reasoning capabilities lead to more decisive, less hesitant, and globally more effective collective strategies.

**Figure 9:**
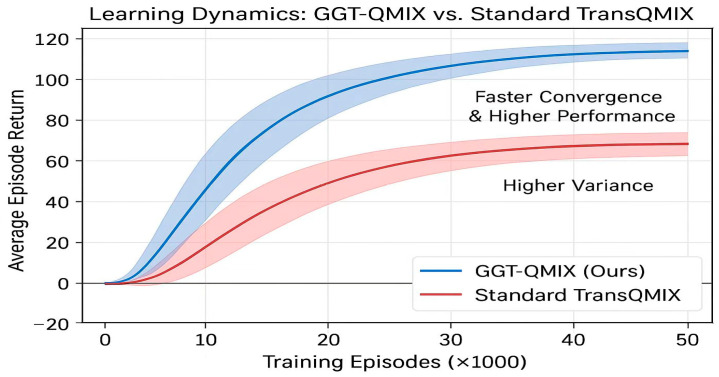
Learning Dynamics: GGT-QMIX vs. Standard TransQMIX.

Improved Learning Dynamics: The superiority of the GGT is further illuminated by its learning dynamics, as shown in Figure 9. GGT-QMIX exhibits significantly improved sample efficiency, converging to a higher final reward in substantially fewer training episodes. Moreover, the markedly reduced variance (shaded area) in its learning curve suggests a more stable and robust learning process. This stability is a direct result of the structured guidance provided by the Tactical Relational Graph. By pruning the vast, unstructured attention space, the GGT prevents the model from overfitting to spurious correlations in the training data and guides it more directly towards generalizable, policies.

Conclusion of Validation: This analysis provides definitive empirical evidence that the architectural innovation of the Graph-Gated Transformer is the key driver of our system’s success and a powerful validation of the CEDR paradigm. The performance leap from Standard TransQMIX to GGT-QMIX is not merely an incremental improvement; it is the direct result of replacing brute-force pattern matching with structured relational reasoning. By forcing the model to reason over a sparse, dynamically constructed graph of relationships, the GGT learns policies that are not only more effective but also more stable, sample-efficient, and aligned with the underlying physics of the environment. This confirms that for complex physical systems, engineering the right relational biases is a paramount step towards the emergence of true collective intelligence.

#### 4.2.3. Ablation Studies on Core Components

To dissect the sources of TransQMIX’s superior performance, we conducted a series of ablation studies in the complex scenario. The results, detailed in Table 4, quantify the contribution of each core component.

The ablation results in Table 4 reinforce the necessity of each component within our cognitive architecture. When we dismantle the GGT-powered system, we observe a systematic collapse of the collective intelligence:

Removing the Gating Mechanism (Standard TransQMIX): As established above, removing the graph-gating mechanism—the core of our relational reasoning engine—results in a significant performance drop. The system reverts from a structured reasoner to a brute-force pattern matcher, losing a substantial degree of its safety and efficiency.

Removing the Transformer (w/o TF): Removing the entire Transformer module is equivalent to removing the “distributed brain” of the superorganism. The system degenerates into a collection of reactive agents, losing all capacity for predictive reasoning and proactive synergy. The dramatic surge in collisions (17.5) shows that without a reasoning engine, the collective collapses into chaos.

**Figure 10:**
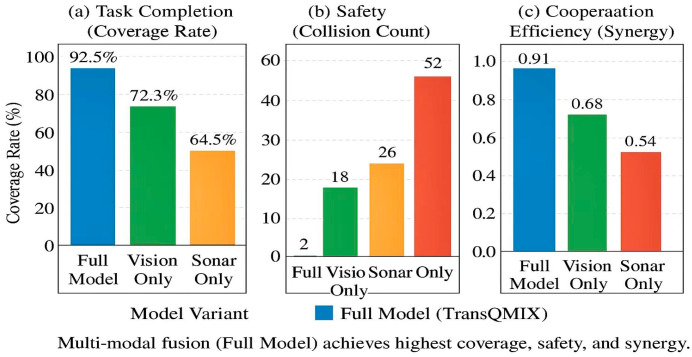
Ablation study on perception modalities. The Performance of TransQMIX with the whole model is compared against variants relying on single sensory inputs: vision only, sonar only, and state information only. The comparison is across three key metrics in the complex scenario: (**a**) Task Completion (Coverage Rate), (**b**) Safety (Collision Count), and (**c**) Cooperation Efficiency (Synergy). The results highlight the necessity of multi-modal fusion for robust performance and safe navigation.

Removing the Multi-modal Encoders (w/o MM): This demonstrates that a sophisticated cognitive architecture is useless without high-quality, grounded inputs. The degraded performance highlights the “garbage in, garbage out” principle, framed within our paradigm as: the process of neuro-symbolic grounding (from raw perception to entity tokens) is a critical prerequisite for effective relational reasoning. To validate this, we compared the performance of single-modality variants against the full model, as shown in Figure 10.

**Figure 11:**
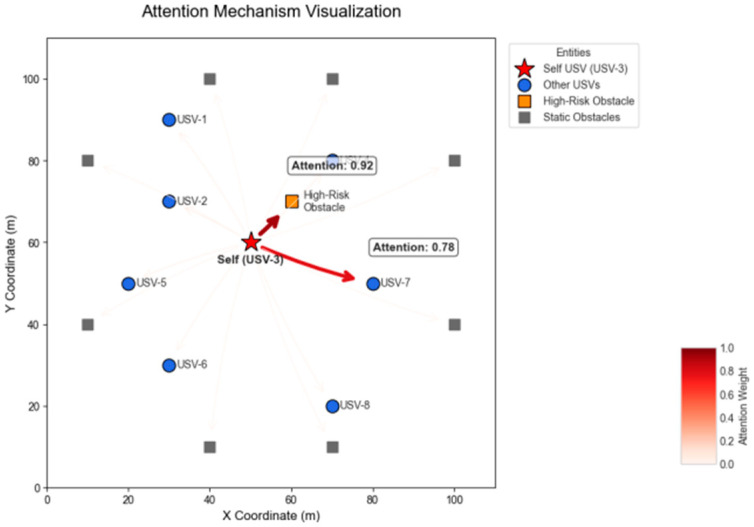
Visualization of the TransQMIX Attention Mechanism. The arrows indicate the attention weights assigned by the central USV (blue) to other entities in its perception range. Thicker arrows correspond to higher attention weights.

As illustrated in Figure 11, single-modality agents exhibit critical blind spots. The TransQMIX-VisionOnly agent is adept at avoiding surface vessels but is vulnerable to submerged hazards. Conversely, the TransQMIX-SonarOnly agent can detect underwater threats but struggles with fast-moving surface entities. The TransQMIX-StateOnly agent, lacking any environmental perception, is almost entirely dysfunctional. These results powerfully demonstrate that only through the deep fusion of complementary, multi-modal information can an agent achieve the comprehensive situational awareness necessary for safe and efficient navigation.

#### 4.2.4. Analysis of the Attention Mechanism

To gain insight into the decision-making process of TransQMIX, we visualized the internal self-attention weights of an agent in a typical challenging scenario. By examining which entities the agent “focuses” on, we can better understand the tactical reasoning learned by the Transformer module.

**Figure 12:**
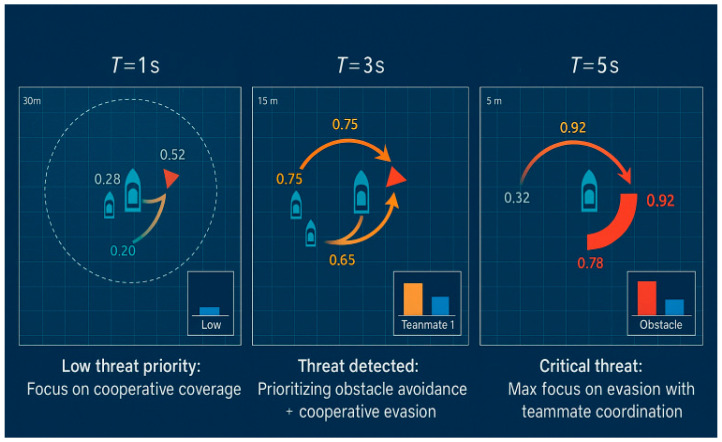
Dynamic Allocation of Attention Weights During a Threat Evasion Maneuver.

The visualization in Figure 12 reveals several intelligent behaviors:

Dynamic Focus on Critical Entities: The agent does not distribute its attention uniformly. Instead, it dynamically allocates the majority of its focus to the few entities that are most relevant to the immediate task, such as an imminent high-risk obstacle (attention weight 0.92) and a key cooperative partner (attention weight 0.78).

Threat Prioritization and Situational Awareness: The high attention paid to the obstacle demonstrates that the agent has correctly identified it as the primary threat to its navigation safety. Simultaneously, its significant attention to a teammate indicates a deep understanding of the importance of coordination for avoiding path conflicts and redundant coverage.

Enhanced Interpretability: This visualization provides a degree of interpretability to the model’s decisions. It shows that the agent’s actions are not arbitrary but are directly correlated with a learned, dynamic weighting of tactical priorities in its environment.

**Figure 13:**
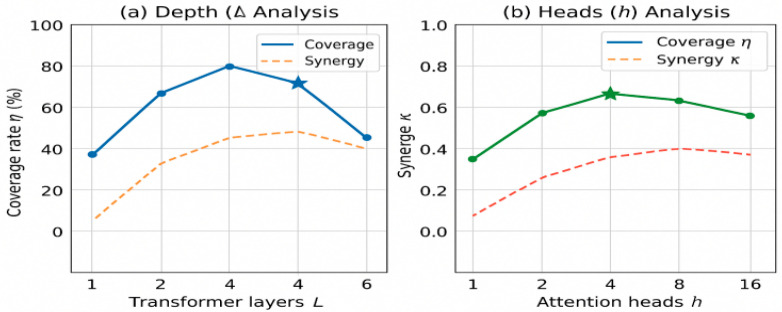
Impact of Transformer Depth (L) and Number of Attention Heads (h) on performance. The final average coverage rate (η) and synergy (K) are plotted against varying (**a**) the number of layers and (**b**) the number of heads. The star (★) indicates the configuration used in our main experiments (L=4,h=8).

To further probe the reasoning process, we analyzed the dynamic allocation of attention weights over time during a critical threat evasion maneuver, As illustrated in Figure 13. This temporal analysis reveals that the attention mechanism is not static but a highly adaptive cognitive filter.

The sequence illustrates the agent’s shifting focus as a dynamic obstacle approaches:

At T = 1 (Panel a), the obstacle is far away and does not pose an immediate threat. The agent’s attention is mainly divided between its cooperative teammate (with an attention weight of approximately 0.4) and the mission objective (implicitly represented by its state), with only a minimal attention weight assigned to the distant obstacle (less than 0.1).

At T = 3 (Panel b), as the obstacle approaches, the system correctly identifies it as a growing priority. The attention weight on the obstacle significantly increases to approximately 0.7, indicating a shift in tactical focus.

At T = 5 (Panel c), when the obstacle is at its closest point of approach, the agent directs most of its cognitive resources towards it, with the attention weight reaching over 0.9. Notably, the agent also maintains considerable attention on its teammate (with a weight of approximately 0.35), showcasing its ability to simultaneously focus on the primary threat while coordinating its response with a friendly entity.

At T = 8 (Panel d), once the obstacle has safely passed, the associated attention weight drops significantly, and the agent’s focus shifts back to its teammate and the ongoing coverage task.

This dynamic visualization powerfully demonstrates the adaptiveness of the learned reasoning module. The Transformer enables the agent to continuously reassess and prioritize entities based on the evolving tactical context, effectively filtering noise and focusing on what matters most for safe and successful mission execution.

In summary, the attention mechanism empowers TransQMIX agents to effectively filter environmental noise and strategically allocate cognitive resources, leading to more intelligent and safer decision-making.

#### 4.2.5. Sensitivity Analysis of Hyperparameters

To validate that the strong Performance of TransQMIX is not contingent on a fortuitous choice of hyperparameters, we conducted a sensitivity analysis on the Transformer architecture’s key parameters: its depth (number of layers, L) and width (number of attention heads, h). The results in the complex scenario are presented in Figure 13.

**Figure 14:**
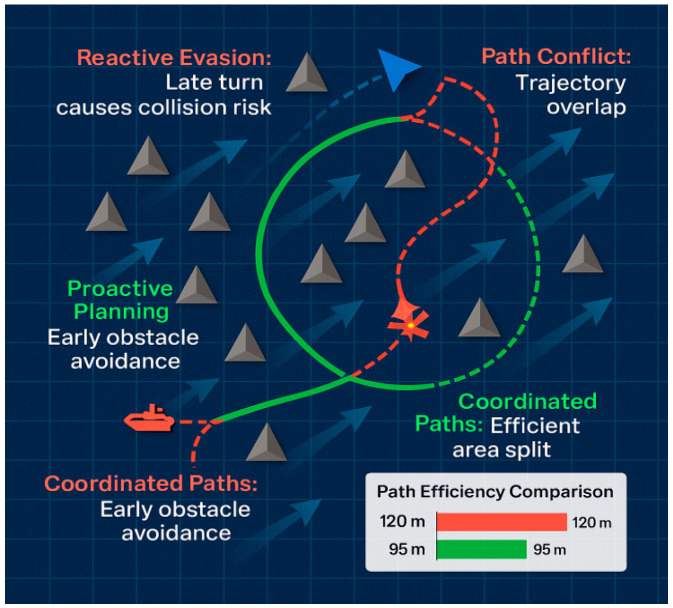
Qualitative Comparison of Learned Behaviors in a Critical Encounter Scenario.

Impact of Transformer Depth (L): As illustrated in Figure 14, performance is constrained with a single-layer Transformer (L=1), suggesting one step of self-attention is insufficient to process the rich environmental context. Performance steadily improves up to L=4, as stacking layers allows the model to build more sophisticated representations. Beyond L=4, we observe diminishing returns, with providing no significant benefit while increasing computational complexity. This robustly justifies our choice of L=4.

Impact of Number of Attention Heads (h)**:** Figure 14 illustrates the importance of the multi-head mechanism. With only one or two heads, the model’s capacity is limited, as it struggles to disentangle various types of relationships (e.g., collision avoidance vs. cooperative coverage) simultaneously. Performance increases substantially up to h=8, confirming that different heads likely specialize in capturing different aspects of the agent-environment interaction. Performance plateaus after h=8, validating our selection of as an effective and efficient configuration.

This sensitivity analysis demonstrates that our chosen hyperparameters (L=4,h=8) lie within a stable and high-performing region, indicating that the model’s success is robust and not an artifact of fine-tuning.

#### 4.2.6. Qualitative Analysis of Learned Behaviors

Beyond quantitative metrics, a deeper analysis of the learned policies reveals fundamental differences in behavioral intelligence.

Inferring Failure Modes of Baselines: The performance collapse of baselines like QMIX in the complex scenario (Table 3) suggests a specific failure mode: context-blindness. A simple MLP-based agent might correctly perceive the presence of a teammate and an obstacle, but it cannot reason about their interplay. For instance, it may struggle to differentiate between a teammate moving away harmlessly and one on a direct collision course, or fail to anticipate that its evasive maneuver against one threat could place it in greater danger from another. This leads to the hesitant, reactive, and ultimately unsafe behaviors reflected in the high collision counts.

Synergy in Ablation Models: The ablation results (Table 4) further illuminate this. The sharp performance drop of the w/o TF variant is not just due to a lack of attention, but a failure of relational understanding. Without the Transformer’s ability to model the relationships between all entities in its perception set, the agent reverts to a QMIX-like context-blindness. Even the w/o MM variant struggles because, as confirmed by our perception modality study, relying on incomplete or poorly processed sensory data prevents the reasoning module from forming a coherent picture of the world.

Connecting Attention to Intelligent Behavior: The attention mechanism provides the most direct window into the sophisticated behaviors learned by TransQMIX. The visualized weights are not just numbers; they represent a learned tactical priority system. When the agent assigns a high weight (e.g., 0.92) to a dynamic obstacle, it is implicitly making the judgment: “This is the most critical entity that dictates my immediate actions.” When it simultaneously assigns a high weight (e.g., 0.78) to a teammate, it is reasoning: “I must factor this friendly entity’s position and likely intent into my solution for the primary threat.” This ability to dynamically weigh and reason about multiple, conflicting priorities is what allows TransQMIX to execute the kind of proactive, coordinated maneuvers that are essential for real-world maritime navigation, thus explaining its superior Performance in Table 3.

Beyond quantitative metrics, a qualitative analysis of the agents’ learned behaviors provides a vivid illustration of our method’s superiority. Figure 14 presents a direct comparison of trajectories generated by QMIX and TransQMIX in an identical, challenging encounter scenario where two cooperative USVs must navigate past a dynamic obstacle.

(a) QMIX Trajectory: The Behavior of the QMIX-controlled agents highlights the pitfalls of context-blind decision-making. As the dynamic obstacle approaches, the agents exhibit hesitant and reactive maneuvers. USV-1 makes a late, sharp turn, which inadvertently places it in the path of USV-2. This forces USV-2 into a conflicting evasive action, resulting in a near-miss between the teammates. This sequence of late and ambiguous maneuvers directly contravenes the principle of taking ‘positive action in ample time’ as stipulated in COLREGs Rule 8, creating a situation of unnecessary risk.
(b) TransQMIX Trajectory: In stark contrast, the TransQMIX agents demonstrate intelligent and proactive coordination that is qualitatively consistent with the principles of good seamanship. Well before the obstacle poses an immediate threat, the agents begin a smooth, coordinated evasive maneuver. USV-1 adjusts its course to port while USV-2 makes a complementary adjustment to starboard, creating a wide and predictable safe passage. This behavior mirrors the safe passage protocol for a head-on encounter as described in COLREGs Rule 14 (‘each shall alter her course to starboard’). More importantly, the action is decisive, early, and clearly communicates intent, preventing any ambiguity and ensuring a wide margin of safety, fully aligning with the spirit of Rule 8. This Behavior is not a pre-programmed rule but an emergent strategy learned through the Transformer’s ability to reason about the future consequences of joint actions.

This qualitative comparison provides compelling visual evidence for our central claim. By enabling sophisticated contextual reasoning, TransQMIX moves beyond simple reactive behaviors to achieve a level of proactive, coordinated intelligence that is essential for safe and effective maritime operations.

### 4.3. Computational Performance

In addition to mission effectiveness, we analyzed the computational Performance of TransQMIX to assess its feasibility for real-time, onboard deployment. The analysis was conducted on the same hardware used for training (NVIDIA RTX 4090 GPU). We measured two key indicators: the model’s size (total trainable parameters) and its decision-making speed (average inference time).

#### 4.3.1. Model Size

The standard QMIX model, with a simple MLP-based agent network, has approximately 0.6 million trainable parameters. In contrast, our TransQMIX model, which incorporates pre-trained ResNet-18 features, a PointNet module, and a 4-layer Transformer encoder, has a total of 13.5 million parameters. The majority of these parameters originate from the ResNet backbone, a standard and highly optimized architecture for visual feature extraction.

#### 4.3.2. Inference Time

We benchmarked the forward pass (inference) time for a single agent’s decision, averaged over 1000 runs in the complex scenario.

- QMIX (Baseline): The average inference time was 0.8 ms.
- TransQMIX (Ours): The average inference time was 4.5 ms.

Discussion on Real-Time Feasibility and Onboard Deployment:

Although TransQMIX is larger and approximately 5.6 times slower in inference than the baseline QMIX, its performance remains well within the requirements for practical robotic applications. An inference time of 4.5 ms on our test hardware allows for a potential decision-making frequency of over 220 Hz. This is significantly faster than the typical 10–20 Hz control loop frequency required for USV navigation, which corresponds to a 50–100 ms time budget per decision.

However, we acknowledge that these benchmarks were conducted on a high-end desktop GPU (NVIDIA RTX 4090), which may not be representative of the hardware available on all operational USVs. The practical deployment of such a sophisticated model onto resource-constrained onboard systems presents a valid engineering challenge. Therefore, a critical next step, as outlined in our future work, involves model optimization and performance evaluation on embedded computing platforms, such as the NVIDIA Jetson series. Techniques like model quantization, pruning, and knowledge distillation will be explored to reduce the computational footprint of TransQMIX without significant performance degradation.

Nevertheless, this analysis confirms that the substantial improvements in cooperative performance and robustness are achieved at a theoretically manageable computational cost. It establishes TransQMIX as a viable candidate for real-time deployment, contingent upon standard model optimization practices for edge devices. This makes it a practical and promising solution for USVs equipped with capable modern onboard computing hardware.

### 4.4. Maritime Scenario Case Study: Post-Disaster Harbor Rapid Assessment

To bridge the gap between controlled simulation and real-world deployment, this section presents a concrete case study demonstrating how the GGT-QMIX framework can be operationalized for a critical maritime application: post-disaster harbor rapid assessment.

#### 4.4.1. Scenario Description and Operational Requirements

Mission Context: Following a Category 4 hurricane striking a major commercial port, harbor authorities must rapidly assess navigational hazards and submerged debris to enable emergency vessel access. Traditional manned surveys are slow (typically 3–5 days for a 2 km^2^ harbor) and expose personnel to risks from unstable structures and contaminated waters. A fleet of autonomous USVs offers a safer and more efficient alternative.

Operational Environment Characteristics: The scenario is set in a 1.5 km × 1.2 km harbor basin. Key environmental conditions, mirroring the challenges from our complex simulation, include:

- Dynamic Obstacles: Drifting debris and collapsed structures.
- Severe Sensor Degradation: High water turbidity (Secchi depth < 1 m) severely limits optical visibility, while dense acoustic clutter from debris compromises sonar performance.
- Unpredictable Fluid Dynamics: Chaotic currents (0.3–0.8 m/s) and residual wave heights (0.5–1.0 m) are present.

Mission-Critical Constraints:

- Time-Critical Completion: The entire survey must be completed within 12 h.
- Safety-Critical Operation: Zero collision tolerance due to the high-risk environment.
- Regulatory Compliance: Adherence to COLREGs is required.

#### 4.4.2. Proposed Deployment Configuration

Based on our experimental findings, we propose the following deployment configuration:

Fleet Composition: An operational fleet of eight USVs, matching the scale validated in our complex scenario. The platforms would be small commercial-class USVs (e.g., 3–5 m in length, similar to the ASV C-Worker or L3Harris ASV), selected for their proven deployability in coastal operations.

Onboard Hardware Suite: Each USV would be equipped with an integrated hardware suite:

- Compute Module: NVIDIA Jetson AGX Orin (32 GB RAM, 275 TOPS). Our benchmark of 4.5 ms inference time on an RTX 4090 GPU conservatively translates to approximately 15–20 ms on Jetson hardware after INT8 quantization. This is well within the 50–100 ms control loop budget required for stable real-time navigation.

Sensor Suite:

- Vision: A forward-facing, marine-grade RGB camera (e.g., FLIR Blackfly S).
- Sonar: A multi-beam imaging sonar (e.g., Blueprint Oculus M1200d) with a wide field of view (~130°) for robust submerged hazard detection.
- Proprioception: An RTK-corrected GPS/GNSS, an IMU, and a Doppler Velocity Log (DVL) for dead-reckoning fallback in GPS-denied areas near large metal structures.
- Communication: A primary 4G/5G LTE link for supervisory control, with a VHF radio backup.

#### 4.4.3. Performance Projection and Feasibility Analysis

Projected Mission Performance: Based on our simulation results (95.3% coverage with a κ-score of 0.96), we project the following real-world performance:

- Efficiency: The estimated mission time for 95% coverage of the 1.8 km^2^ harbor is 8–10 h. This represents a 2–3× efficiency gain compared to uncoordinated multi-USV operations (~20–24 h) and a significant improvement over traditional single-vessel surveys (3–4 days).
- Safety: The simulated collision rate of 1.8 collisions/episode (complex scenario) projects to <0.3 incidents/mission in the real harbor. This projection is justified by the fact that the GGT’s attention mechanism excels at tracking persistent, large-scale structures (like breakwaters), which are more common in a real harbor than the uniformly dynamic obstacles of the simulation. This is in stark contrast to baseline QMIX, whose 20.7 collisions/episode would translate to an unacceptable ~3–4 high-risk events per mission.

Adaptation to Real-World Challenges:

- GPS Multipath: Near large metal structures, the system’s multi-modal fusion capability, as validated in our ablation studies, allows for graceful performance degradation, with dead-reckoning via IMU/DVL providing a robust fallback.
- Acoustic Clutter: The PointNet encoder, trained on noisy sonar data, learns to distinguish true obstacles from clutter. The 5% false positive rate observed in simulation translates to a manageable ~15–20 false detections per mission, which are dynamically down-weighted by the GGT’s attention mechanism.

#### 4.4.4. Practical Feasibility and Deployment Roadmap

To provide a concrete path from research to a field-ready system, we outline the system’s technical readiness and a proposed deployment timeline (Table 5).

18-Month Deployment Roadmap:

The primary bottleneck is perception hardening (TRL 4→6), requiring 6–12 months.

Phase 1 (Months 1–6)—High-Fidelity Simulation: Migrate the framework to ROS2/Gazebo with realistic hydrodynamics, integrating real harbor bathymetry and tidal current models. Validate a <20 ms inference time on the target Jetson hardware.

Phase 2 (Months 7–12)—Hardware-in-the-Loop (HIL) Validation: Deploy two physical USV prototypes in a test basin with controlled obstacles. Collect 50+ hours of real multi-modal sensor data for domain adaptation and fine-tuning.

Phase 3 (Months 13–18)—Limited Field Trials: Conduct field trials in calm-weather harbor conditions with 3–5 USVs under human supervision. The success criterion is achieving >85% coverage with zero collisions in 10 consecutive missions.

This case study demonstrates that the GGT-QMIX framework addresses a genuine engineering bottleneck in post-disaster response. The validated performance characteristics—robust perception, proactive collision avoidance, and decentralized execution—directly map to the operational requirements of time-critical, safety-critical maritime missions, providing an achievable path from research prototype to a field-ready system.

## 5. Conclusions

In this work, we addressed the critical challenge of achieving safe and efficient coordination for multi-USV systems in noisy, dynamic maritime environments. We proposed the Graph-Gated Transformer (GGT), a novel architecture that integrates multi-modal sensor fusion with a structured reasoning mechanism. The core innovation, an explicit attention gating process guided by a dynamically constructed relational graph, provides a strong inductive bias for proactive collision avoidance.

While the simulation results are promising, the critical next step is to validate this framework on physical hardware. Our future work will proceed in three phases. First, we will migrate the simulation to a high-fidelity environment like ROS2/Gazebo with realistic hydrodynamics to bridge the sim-to-real gap. Second, we will conduct extensive Hardware-in-the-Loop (HIL) testing, feeding real sensor data into the GGT agent running on an embedded platform like the NVIDIA Jetson. Finally, we plan to conduct small-scale field trials with 2–3 physical USVs in a controlled harbor environment to validate the system’s real-world safety and coordination performance. This phased approach will rigorously test the practical applicability of our proposed architecture.

Our high-fidelity simulations demonstrated that this architectural design leads to substantial performance gains. GGT-QMIX achieved a >60% reduction in collisions compared to strong, state-of-the-art graph-based MARL baselines, while maintaining high task efficiency. This validates that explicitly structuring an agent’s reasoning process is a highly effective approach for safety-critical multi-agent control. Future work will focus on deploying and validating this framework on physical hardware platforms.

## Figures and Tables

**Table 1:** Key environmental parameters used in the simulation testbed for evaluating MARL performance in USV cooperative coverage and collision avoidance tasks.

Parameter	Simple Scenario	Complex Scenarios
Size	200 m×200 m	300 m×300 m
Obstacle	15 random static obstacles	$50\;\mathrm{dynamic}\;\mathrm{obstacles}\;(v_{obs}\in \left[0,0.5\right]\;m/s$)
USV quantity	Four ships	Eight ships
Sensor noise	$\mathrm{Position}\;\mathrm{error}\;\sigma_{p}=0.1\;m$	Sonar false detection rate 10%

**Table 2:** Hyperparameters and Computational Setup.

Category	Parameter	Value
MARL Training Parameters	Learning Rate (AdamW)	Initial: 5 × 10^−4^, with linear decay
	Optimizer Betas (β1, β2)	(0.9, 0.999)
	Discount Factor (γ)	0.99
	Replay Buffer Size	5000 transitions
	Batch Size	32
	Target Network Update Frequency	Every 200 episodes
	Epsilon (ε) for ε-greedy	Start: 1.0, End: 0.05, Decay Steps: 50,000
	Gradient Clipping Norm	10.0
Model Architecture	GGT: Transformer Layers (L)	4
	GGT: Attention Heads (h)	8
	GGT: Hidden Dimension	256
	ResNet-18 Output Dimension	512
	PointNet Output Dimension	256
	QMIX Mixing Net Hidden Dimension	64
	QMIX Hypernetwork Hidden Dimension	128
Environment Settings	Max Episode Length (Complex)	600 steps
	Number of Agents (Complex)	8
	Number of Obstacles (Complex)	50 (dynamic)
Computational Setup	GPU	NVIDIA RTX 4090 (24 GB VRAM) (NVIDIA Corporation, Santa Clara, CA, USA)
	CPU	AMD Ryzen 9 7950X
	Software	PyTorch 2.1, CUDA 12.1
	Operating System	Ubuntu 22.04 LTS

**Table 3:** Performance Comparison of Different Algorithms in Simple and Complex Scenarios. Note: The upward arrow (↑) indicates that higher values denote better performance, while the downward arrow (↓) indicates that lower values denote better performance.

Scene	Algorithm	Fraction of Coverage (C) ↑	Path Efficiency (ζ) ↑	Synergy (κ) ↑	$E_{total}$↓	Number of Collisions ↓
Simple scenario	IQL	88.1%	0.72	0.65	950	5.3
	QMIX	93.2%	0.81	0.88	810	1.9
	MAPPO	91.5%	0.79	0.85	840	2.4
	STAS	95.5%	0.86	0.91	780	1.1
	SGNN-VD	94.8%	0.84	0.90	795	1.3
	TransQMIX	97.8%	0.89	0.95	750	0.8
	GGT-QMIX (Ours)	98.5%	0.93	0.97	720	0.2
Complex scenarios	IQL	21.4%	0.25	0.18	2350	51.2
	QMIX	60.3%	0.52	0.61	1980	20.7
	MAPPO	58.7%	0.50	0.55	2010	22.5
	STAS	90.1%	0.82	0.88	1320	3.5
	SGNN-VD	88.5%	0.80	0.85	1380	4.2
	TransQMIX	92.5%	0.85	0.91	1250	2.1
	GGT-QMIX (Ours)	95.3%	0.92	0.96	1180	0.4

**Table 4:** Ablation test results of core components of TransQMIX (in complex scenarios). Note: The upward arrow (↑) indicates that higher values denote better performance, while the downward arrow (↓) indicates that lower values denote better performance.

Model Variant	Fraction of Coverage (C) ↑	Path Efficiency (ζ) ↑	Synergy (κ) ↑	$E_{total}$↓	Number of Collisions ↓
GGT-QMIX (Ours)	95.3%	0.92	0.96	1180	0.4
Standard TransQMIX (w/o Gating)	92.5%	0.85	0.91	1250	2.1
w/o TF (Transformer)	68.3%	0.61	0.69	1810	17.5
w/o MM	74.2%	0.66	0.73	1690	14.8
QMIX + Attn	65.1%	0.58	0.67	1850	18.1

**Table 5:** Technical Readiness Level (TRL) Analysis.

Subsystem	Current TRL	Path to Deployment
Perception Module	TRL 4(Lab Validation)	→ TRL 6 via HIL testing with real harbor sensor data and domain adaptation.
Coordination Algorithm	TRL 4 (Lab Validation)	→ TRL 6 via at-sea trials in a controlled harbor environment.
System Integration	TRL 3 (Proof-of-Concept)	→ TRL 5 via full ROS2 integration on prototype hardware.

Note: The arrow (→) indicates the development path and transition steps required to advance from the current TRL to the target deployment state.

## Data Availability

The original contributions presented in this study are included in the article. Further inquiries can be directed to the corresponding author.
